# Central Neuraxial Blockade-Assisted External Cephalic Version in Reducing Caesarean Section Rate: Systematic Review and Meta-Analysis

**DOI:** 10.1155/2009/718981

**Published:** 2009-12-23

**Authors:** Ibrahim Bolaji, Lillian Alabi-Isama

**Affiliations:** ^1^Family Services Division, Department of Obstetrics and Gynaecology, Hull York Medical School (HYMS), Diana Princess of Wales Hospital, Northern Lincolnshire and Goole Hospitals NHS Foundation Trust, Scartho Road, Grimsby DN33 2BA, UK; ^2^Clinical Research Fellow, Division of Surgery, Oncology, Reproductive Biology and Anaesthetics, Imperial College, Hammersmith Campus, Du cane Road, London W120NN, UK

## Abstract

We review the medical literature on the success, safety and economic value of central neuraxial blockade-assisted (CNB) external cephalic version from randomized controlled studies identified from 1951 to 2009. The result showed that more women had successful ECV with regional anaesthesia with corresponding reduction in caesarean section rate. They were 1.5 times more likely than women not receiving anaesthesia to have a successful ECV. The number to treat is six women needed to receive anaesthesia for 1 baby to be turned from breech to cephalic presentation. Feto-maternal morbidity was not increased in the CNB-aided group consisting of only transient bradycardia. Although the appropriate amount of force for safe version has not been quantified, there was no report of uterine rupture despite removal of these patients from “excessive force-pain biofeedback loop” induced through motor nerve blockade. We can attribute 30% of cost savings amounting to £42,150.00 directly to CNB using the most up to date Health Resource Group Code (HRG4). The initial results are encouraging but until the benefits and safety of CNB-aided ECV are substantiated by large randomized, blinded controlled trials, this practice cannot be universally recommended.

## 1. Introduction

The problem of rising caesarean section is one of paramount importance and continues to be of increasing interest and concern. This increase is not limited to Europe [1] but global [2] and although this increase involves principally the lower segment procedure, classical caesarean section has not been excluded [3]. Despite the growing national and international trend some units still maintain a relatively low caesarean section rate (CSR) (Table 1).

One suggested reason for the rise is increasing requests by women for caesarean section (CS) in the absence of clear medical indications such as placenta praevia and arguably, breech presentation, or previous caesarean section [4, 5].

Several authors have suggested a number of strategies for reducing CSR [1, 6–8], particularly “primary C-section,” including external cephalic version (ECV). Turning the fetus to the cephalic/vertex position by external manipulation has been identified as the only clinical intervention with demonstrated grade A evidence of reducing CS overall [6]. 

Approximately 4% of term singleton babies present as a breech [9]. This accounts for nearly 20% of primary CS and this rate is rising [1]. This increase is partly due to “defensive obstetrics” and has been more dramatic following the publication of the term breech trial [10]. Consequently, a trial of ECV at term has become increasingly popular. The majority of women would prefer a vaginal birth (VB) [11] although most would choose CS if there is a medical indication [12]. For single fetus in breech presentation, CS has been shown to be safer for the fetus than VB [10, 13] and the majority now recommend CS for term breech presentation [14]. Although the risks associated with operative delivery are low, it is not without fetomaternal incidents and CS remains the largest contributing factor to the incidence of maternal morbidity and mortality in the pueperium [15]. Operative delivery quadruples the risk of severe morbidity compared to VB [16–18]. 

 The rationale for ECV is to reduce the incidence of breech presentation at term, the associated risks of vaginal breech delivery, and also the risk of avoidable caesarean section [7]. Success rates for ECV have been variable, ranging from 35%–86% with an average of 58%, and a number of strategies have been proposed to improve this, thereby reducing the CSR. 

 Tocolytics have been used in this regard in the last three decades [19], with initial studies reporting success rates of about 60%–80% [19, 20]. Terbutaline was found to be the most commonly used beta-sympathomimetic agent to achieve tocolysis prior to ECV. 

 A recent Cochrane Database of systemic reviews on interventions to help ECV concluded that routine tocolysis appears to reduce the failure rate of ECV at term [21]. Although the reduction of noncephalic presentations at birth was not statistically significant, there was, however, a reduction in the number of CSs [21].

Nonmedical interventions to improve spontaneous version rate have been described by several authors. They include postural techniques, entailing relaxation with the pelvis in an elevated position (knee-chest position assumed with full bladder) [22], “Indian Version” [23], Vibroacoustic stimulation for midline spine position [24], transabdominal amnioinfusion with warmed saline [25], acupuncture [26], and complementary medicine in form of moxibustion. The latter is a traditional Chinese medicine involving the burning of a herb, *Artemisia vulgaris*, close to the skin to induce warming sensation [27]. There is, however, insufficient evidence to support these methods. Other alternatives including the use of hypnosis and acupuncture or moxibustion to the acupuncture point bladder 67 (BL67), located at the tip of the fifth toe [26], have been poorly researched. A recent randomized controlled trial on moxibustion for breech version [27] showed no beneficial effect. Despite this lack of proven effectiveness, women had positive opinions of the intervention.

## 2. Method

This paper reviews the medical literature to determine the success, safety, and economic value of regional anaesthesia-assisted ECVs. 

 The inclusion criteria include clinical trials evaluating ECV success using both randomized and nonrandomized trials. The interventions studied were all methods of maternal anaesthesia including general anaesthetic, central neuraxial anaesthesia (CNB), or analgesia used to aid ECV. 

 The primary outcome was the proportion of participants with a successful external cephalic version at the end of the attempts. Secondary outcomes included fetal presentation at delivery, method of delivery, fetal morbidity and mortality, and maternal morbidity. 

 Articles were identified in searches using the following electronic databases: MEDLINE (MEDLARS OnLine), Cochrane, and RCOG using DIALOG Information Retrieval Service covering the period 1960–2009 under the following medical subject heading key words: *external cephalic version and anaesthesia or analgesia *in either the heading or text words*.*

 Hand searches of the articles identified within references were conducted as well as searches from the International Journal of Obstetric Anaesthesia and the published abstracts of the annual meetings of the American Society of Anaesthesiologists and the Canadian Anaesthesia Society. All attempts were made to identify relevant data on this topic. 

 Two reviewers (L. AI and I. IB) inspected titles and abstracts of all the references retrieved from the search to select all potentially relevant studies. Studies that met the inclusion criteria were categorized. Each potentially relevant study was then reviewed to identify randomized control trials. 

 Results from the randomized trials were combined using random effects model. The primary outcome which is successful ECV was expressed in relative risk (RR) with 95% confidence intervals. An RR of greater than 1.0 would indicate the proportion of women with successful ECV with anaesthesia over those without anaesthesia. Statistical significance was defined as a *P* value <.05 and if the 95% confidence interval (CI) did not include 1.0. Numbers needed to treat (NNT) are the inverse of the Absolute Risk Reduction (NNT = 1/ARR) and absolute risk reduction (ARR) is the experimental event rate-control event rate. 

 Twenty nine studies were identified from initial literature search and reviewed. The articles ranged in publication from 1951 to 2009. Of these, 22 were nonrandomized prospective and retrospective studies. Only seven articles were randomized controlled trials including two studies comparing the use of epidural anaesthesia [28, 29] with no anaesthesia, four studies compared the use of spinal anaesthesia [30–33] with no anaesthesia, and one study compared the use of combined spinal and epidural anaesthesia [34] with no anaesthesia. Six of the 7 studies were conducted in the US, the seventh study was conducted in Israel, and all seven studies were published between 1997 and 2009. The study by Delisle et al. [32] was published in abstract form with limited study information available. There were 681 women participating in the seven studies with 339 women receiving either epidural or spinal anaesthesia, 47 women receiving intravenous fentanyl, and 295 receiving no anaesthetic.

## 3. Effectiveness of Central Neuraxial Blockade-Assisted External Cephalic Version

There has been renewed interest in the practice of central neuraxial blockade-assisted ECV at term especially in the USA. Central neuraxial blocks are a group of anaesthetic techniques which include epidurals, spinals, and combined spinal epidurals (CSEs). All are invasive techniques involving injection of pain relieving drugs into the vertebral canal (spinal) and epidural space (epidural) and requiring a needle to be placed close to the central nervous system. CNB has the potential to provide patients with optimal pain relief but can also lead to patient harm. The 3rd National Audit Project of the Royal College of Anaesthetists [35] provides reassuring results for the use of this technique. 

 It is known that ECV does not currently play a significant role in the management of breech presentation and that epidural-assisted ECV might improve success rates. Preterm ECV is a subject of an ongoing international multicentre randomized study [36] and is not considered in this review. 

 ECV is at the very least uncomfortable, if not overtly painful and is associated with maternal anxiety. Maternal discomfort and anxiety are associated with higher rates of failure of ECV and for that reason regional anaesthesia has been proposed to facilitate ECV. The rationale for the inclusion is that whilst tocolytic agent provides uterine relaxation, CNB provides abdominal muscular relaxation through motor nerve block hence its effects on maternal pain, muscle tone, and anxiety. General anaesthesia has been used in the past for ECV but was abandoned following reports of fetal mortality, which in some series reached 1% [37]. 

 A retrospective study in the setting of previously failed ECV attempts by Cherayil et al. [31] showed that the success rates in nulliparous women reattempted with spinal and epidural anaesthesia were 4/5 (80%) and 5/6 (83%), respectively. Success rates in multiparous women reattempted with spinal and epidural anaesthesia were 1/1 (100%) and 3/3 (100%) (*P* = NS). High ECV success rates were noted in nulliparous (9/11 [82%]) and multiparous (4/4 [100%] (*P* = .36) women, respectively. Overall, 8/9 (89%) and 5/6 (83%) (*P* = NS) ECV attempts were successful with epidural and spinal anaesthesia, respectively. 

 In a retrospective cohort study by Carlan et al. [38], ECV at term was successful in 59% of 32 women with epidural analgesia, and 24% of 37 women without anaesthesia. Albeit a retrospective analysis and limited as well by a relatively small number of patients, the study shows an encouraging increase in the success rate for ECV performed with epidural analgesia. This is an impressive difference, because in the epidural group there were relatively more versions performed by nonconsultant grade and more patients with advanced cervical dilatation or even in labour. Both of these factors would be expected to decrease success in the study group. In addition, of the 28 patients who failed a first attempt at version without anaesthesia, 7 patients had successful versions when a second attempt was performed with CNB. 

 In an uncontrolled study, ECV under epidural analgesia was successful in nine (56%) of 16 women in whom initial attempts had failed [39, 40].

 Several randomized controlled trials have compared the use of regional anaesthesia with no anaesthesia before performing ECV. Weiniger et al. [30], in a randomized control trial of ECV using regional anaesthesia amongst nulliparous women showed that ECV was more successful in the women who were randomized to receive spinal anaesthesia (24 of 36 [66.7%] compared with 11 of 34 [32.4%], who did not receive spinal anaesthesia (*P* = .04). Fifteen women in the control group had unsuccessful version due to pain that did not allow a sufficient ECV attempt and all fifteen were offered subsequent spinal anaesthesia. Eleven of them had successful ECV under spinal anaesthesia. Sullivan et al. [34] randomized 48 women to receive combined spinal and epidural anaesthesia (CSE) and 47 women to receive intravenous fentanyl (SYS) and showed that a success rate of ECV was 47% with CSE and 31% in the SYS group (*P* = .14). There was no difference in the rate of successful ECV or vaginal delivery with CSE compared to intravenous fentanyl analgesia. Pain scores were lower and satisfaction higher with CSE analgesia, and median time to fetal heart rate reactivity was shorter in the CSE group. 

 A meta-analysis by Marcarthur et al. [41] compared four prospective randomized studies measuring the success rate of ECV performed using neuraxial analgesia and described an overall success rate of 50%. They found that more women had successful ECV if they received regional anaesthesia (119/238; 50%) compared to women who did not receive anaesthesia (82/242; 34%). 

 There was discordance between the trials included in our review (Table 2). ECV failure was significantly reduced in the two trials using epidural anaesthesia [28, 29] but no difference was found in the trial by Dugoff et al. [31]. Schorr et al. [29] demonstrated a significant beneficial effect of anaesthesia in both primary and secondary outcomes. Only one women in the control group reverted from a cephalic version to a breech after successful ECV compared to none in the anaesthetic group. The number of caesarean sections in the control group was more than those in the treatment group. In the study by Macusso et al. [28], there was no statistical difference between the groups in gestation at the time of procedure, placental location, fetal lie, estimated fetal weight, or amniotic fluid index but ECV was successful in 32 of 54 women (59%) with epidural anaesthesia compared with 18 of 54 (33%) with no anaesthesia (RR 1.78, 95% CI 1.15, 2.75, *P* < .05). Vaginal delivery occurred in 29 of 54 women (54%) in the treatment group and 16 of 54 women (30%) in the control group (RR 1.81, 95% CI 1.14, 2.92, *P* < .05). The study by Weiniger et al. [30] showed that successful ECV occurred among 24/36 (66.7%) women randomized to receive spinal analgesia compared with 11 of 34 (32.4%) without anaesthesia (RR 2.06, 95% CI of 1.25, 3.43; *P* < .05). This contradicted the spinal study by Dugoff et al. [31] where there was no statistical difference between the anaesthetic {22/50*  *(44.0%)*  P* = .03} and no anaesthetic group {(22/52*  *(42.3%)*  P* = .1}.

## 4. Safety of Central Neuraxial Blockade-Assisted External Cephalic Version

One major concern has traditionally been that if the patient is removed from “excessive force-pain biofeedback loop,” then there is potential risk that ECV facilitated by regional block could lead to use of excessive force. This may result in increased incidence of abruption, Rh-isoimmunisation, uterine rupture, cord prolapse, and other serious conditions. Fetal morbidity was not increased in the group that had regional anaesthesia consisting only of a transient bradycardia (9.9%; 26/263). One attempt in both spinal and epidural group was associated with fetal bradycardia needing caesarean section with APGAR scores of 8 and 9 at 1 and 5 minutes, respectively [42]. The outcome of fetal morbidity was not calculated as there was no fetal or neonatal death. Although the appropriate amount of force for safe version has not been quantified, there have been no reports of uterine rupture during attempted ECV under neuraxial analgesia (Table 3). 

 In our unit, we adopt “the rule of 3” which consists of three criteria for stopping the ECV (Table 4). The procedure is discontinued if the woman reports undue discomfort, if the fetal heart tones are nonreassuring or there is unsuccessful version after three manoeuvres. 

 Stephen Pratt [44, 45] has argued in favour of epidural use for ECV. He suggested that the use of continuous fetal monitoring and real time ultrasound scan is now routine during ECV, allowing the clinician to closely monitor mother and baby, thereby greatly decreasing the risk of catastrophic events. 

 On the other hand, there is the risk of epidural anaesthesia [35] such as dural puncture and headaches, haematoma, and other neurological complications. One plausible way of reducing the risk of regional anaesthesia is to delay this procedure until the onset of established labour when epidural can be offered for pain relief but the downside is the membranes may have ruptured already hence, reducing the success of the ECV attempt. Neuraxial anaesthesia can be used selectively in women with failed attempts or very anxious women in an environment where Caesarean delivery is immediately possible if the version fails or significant complication occurs monitored by cardiotocograph.

## 5. Cost Effectiveness of Central Neuraxial Blockade-Assisted External Cephalic Version

We use the new Health Resource Groups [46] (HRG) for cost calculation. HRGs are standard groupings of clinically similar treatments which use common levels of healthcare resource. They enable the comparison of activity within and between different organisations with the National Health Service. 

 National tariff is the national price for each HRG for each patient spell in hospital. There are separate tariffs for elective and emergency care and providers are compensated for unavoidable regional cost differences. Their use as consistent “units of currency” supports standardised healthcare commissioning across the service. The previous version of HRGs (v3.5) has been used since 2003 but lately the Casemix service has introduced a major revision, HRG4 [47], which was commissioned by the Department of Health (DoH), to support the policy of payment by results [48] (PbR) from 2009-10 for payment. 

 Using the most up to date HRG4 code, the cost of HRG NZO3A (national tariff for CS 19 years and over) minus HRG cost NZO1B (national tariff for normal delivery 19 years and over without complications) for each successful version would generate an estimated savings of £1,405.00 (Table 5). The use of CNB adds approximately 30 successful versions per 100 attempts (i.e., from 25%–30% success rate up to 55%–60%) [45]. We can attribute 30% of this cost saving or £42,150.00 pounds sterling directly to the CNB. In the new HRG version 4 there is no individual cost for epidural as this is absorbed into the cost of the maternity event itself. If we adopt a conservative estimate of 15% (as not all successful versions will deliver vaginally) or an additional 15 successful versions/100, the cost saving per regional block would be £21,750.00. This is similar to results of previous studies [44, 49]. 

 If delivery is planned immediately after attempted ECV, the epidural could be used for labour analgesics if the version is successful or for operative delivery if it fails. The later practice would limit any additional cost attributable to the version, particularly those related to nursing or hospital charges which would make the cost–benefit analysis even more favourable. There is currently no incentive such as financial benefits for units pursuing strategies to reduce Caesarean section as the hospitals get paid for services rendered under the PbR. 

 It is anticipated that there would be a commitment by the DoH to make a proportion of providers' income conditional on quality and innovation, through the Commissioning for Quality and Innovation [50] (CQUIN) payment framework.

For the acute sector, this would be through a CQUIN scheme linking payment to specific locally determined goals. Our trust and Yorkshire and Humber NHS organisations are one of the front runners to sign up to delivery of a regional set of quality indicators as the mechanism for delivering the nationally set CQUIN policy. This option will be available in 2009/10 as an interim stage.

## 6. Conclusion

This systematic review compared seven randomized controlled trials and found more women had successful ECV if they received regional anaesthesia (174/339; 51.3%) than women who did not receive anaesthesia (103/295; 34.9%). Thus women receiving anaesthesia were 1.5 times more likely than women not receiving anaesthesia to have a successful ECV (95% CI 1.22–1.77). Number needed to treat (NNT) is 6 women needed to receive anaesthesia for 1 baby to be turned from a breech to cephalic presentation. 

 It showed CNB-aided ECV as an additional tool for women with breech-presenting fetus. When successful, it may reduce CS in the index pregnancy and may allow for normal deliveries in subsequent pregnancies without the risk of trial of scar or placental accretism. A limitation of all the trials is that relatively few women have been involved in the randomized control trials comparing both groups; so estimates of maternal and fetal risks with CNB cannot be quantified. Furthermore, the obstetricians were not blinded to the presence or absence of anaesthesia in these trials. This is bound to influence the operators view to pursue a successful version as well as operators own bias. In the reports of Delisle et al. [32] and Hollard et al. [33], the method of random allocation was not specified. 

 There is no optimal gestational age for which this method could be applied. While delivery immediately following ECV would make the cost benefit analysis more favourable, there is concern that waiting until term to attempt ECV will potentially reduce the success rate and risk onset of spontaneous labour which also reduces success rate or may even be a relative contraindication to attempt ECV. 

 There was no information about the most important outcome which is avoidance of C-section. Not every successful version will deliver vaginally and some may revert while others may require intrapartum CS as high as 16.9% [51]. Because of conflicting results, the use of regional anaesthesia for facilitating external cephalic version cannot be recommended at this stage. A larger randomized, blinded (which would be difficult if not impossible) controlled trial is required to evaluate the safety and success of this practice.

## Figures and Tables

**Table 1:** Eight-year review of caesarean sections-Diana, Princess of Wales Hospital, Grimsby UK^⊥^.

Year	Deliveries	Babies	Caesarean section		Emergency C-section^⊥^		Elective C-section^⊥ ⊥^	
			N	%	N	%	N	%
5-Year review*								
2001	1951	1979	362	18.60%	203	11%	159	8%
2002	1973	1992	344	17.40%	194	10%	150	8%
2003	2069	2106	387	18.70%	221	11%	166	8%
2004	2235	2261	405	18.10%	238	11%	167	8%
2005	2350	2384	387	16.40%	258	11%	129	6%
**Total**	**10578**	**10722**	**1885**	**17.84%**	**1114**	**10.80%**	**771**	**7.60% **
8-Year review**								
2006	2420	2458	413	17.10%	233	10%	180	8%
2007	2458	2535	450	18.10%	240	10%	210	8%
2008	2508	2577	415	16.30%	213	8%	202	3%
**Total**	**17964**	**18292**	**3163**	**17.59%**	**1800**	**10.25%**	**1363**	**7.13%**

^⊥^Diana, Princess of Wales Hospital, Grimsby serves a fairly stable predominantly working class population of about 91000 in the Northeast Lincolnshire, UK. ^⊥ ⊥^C-Section-Lower segment caesarean section. *Five-year review was from January 1, 2001 to December 31, 2005. ** Eight-year review was from January 1, 2001 to December 31, 2008.

**Table 2:** Success rates of central neuraxial block-aided ECV-discordant randomized trials.

Study	*Gestational age	Treatment (%)	Control (%)	RR (95% CI)
*EPIDURAL*				
Schorr et al. [29]	>37 weeks	24/35 (68.6)	11/34 (32.4)	2.12 (1.24, 3.62)
Mancuso et al. [28]	>37 weeks	32/54 (59.3)	18/54 (33.3)	1.78 (1.15, 2.75)
*Total *		*89 *	*88 *	*1.90 (1.38, 2.64)*
*SPINAL*				
Weiniger et al. [30]	>37 weeks	24/36 (66.7)	11/34 (32.4)	2.06 (1.25, 3.43)
Dugoff et al. [31]	38 weeks	22/50 (44.0)	22/52 (42.3)	1.04 (0.67, 1.62)
Delisle et al. [32]	Unknown	41/99 (41.4)	31/102 (30.4)	1.36 (0.94, 1.98)
Hollard [33]	>37 weeks	9/17 (52.9)	10/19 (52.6)	1.01 (0.54, 1.82)
*Total *		*202 *	*207 *	*1.31 (1.04, 1.66)*
*^ ⊥ ^CSE*				
Sullivan [34]	>36 weeks	22/48 (45.8)	18/47 (38.3)	1.19 (0.74, 1.92)
*Overall Total *		**339 **	**342**	**1.45 (1.21, 1.72)**

*Gestational age when ECV was performed. ^⊥^CSE combined spinal and epidural.

**Table 3:** Summary of Central Neuraxial Blockade-Assisted ECV Trials.

Author/Year	Type of Study	Subjects	Success of ECV	Complications
Carlan et al. [38] (1994)	Retrospective chart review at >36 weeks	32 epidural; 37 control	59% success and 54% vag del; 24% and 21% in control	7 bradycardia, 1 abruption with epidural; 2 and 1 in control
Schorr et al. [29] (1997)	Prospective randomized at >37 weeks	35 epidural; 34 control	69% success; 66% vaginal delivery in epidural group; 32% and 31% in control	NA
Dugoff et al. [31] (1999)	Prospective randomized	50 spinal; 52 control	44% success in spinal group; 42% control	11 bradycardia, 4 hypotension with spinal; 6 bradycardia, 1 abruption, 4 patient pain in control
Mancuso et al. [28] (2000)	Prospective randomized at >37 weeks	54 epidural; 54 control	59% success and 54% vag del in epidural group; 33% and 30% in control (*P* < .05)	4% bradycardia in epidural group; 6% in control
Birnbach et al. [43] (2001)	Prospective open at >36 weeks	20 received spinal analgesia; 15 did not	80% success in spinal group; 33% in control	Fetal bradycardia requiring section in 1 pt. in control group
Delisle et al. [32] (2001)	Prospective randomized	99 spinal; 102 control	41% success in spinal group; 30% control	Unknown
Hollard et al. [33] (2003)	Prospective randomized	17 spinal; 19 control	52.9% success in spinal group; 52.6% control	1 placenta abruption in spinal group
Weiniger et al. [30] (2007)	Prospective randomized at >37 weeks	36 spinal; 34 control	66.7% success in spinal group; 32.4% control	2 transient bradycardia, 7 hypotension
Sullivan et al. [34] (2009)	Prospective randomized at ≥36 weeks	22 CSE^⊥^; 18 IV fentanyl	45.8% success in CSE group; 38.3% IV fentanyl group	1 bradycardia in each group needing EMCS*

^⊥^ CSE combined spinal and epidural. *EMCS Emergency Caesarean Section.

**Table 4:** Grimsby ECV rule of three^±^.

*Criteria for stopping external cephalic version (ECV)*	
1	Undue Maternal Discomfort
2	Non-reassuring Fetal Heart Tones/Patterns
3	Unsuccessful version after three manoeuvres

^±^ECV is discontinued if one of the above 3 criteria occurs.

**Table 5:** Delivery Event National ^±^PbR Tariffs 2008-09 & 2009-10^⊥^.

2008-09		
HRG V3.5	Description	**Cost in £
N06	Normal Delivery with CC^±^	996
N07	Normal Delivery without CC	996
N08	Assisted Delivery with CC	2029
N09	Assisted Delivery without CC	1422
N10	Caesarean Section with CC	3077
N11	Caesarean Section without CC	2198

2009-10		

HRG V4	Description	**Cost in £

NZ01A	Normal delivery 19 years and over with CC	1881
**NZ01B**	**Normal delivery 19 years and over without CC**	**1174**
NZ01C	Normal delivery 18 years and under with CC	1921
NZ01D	Normal delivery 18 years and under without CC	1177
NZ02A	Assisted delivery with CC	2288
NZ02B	Assisted delivery without CC	1728
**NZ03A**	**Caesarean Section **19** years and over**	**2579**
NZ03B	Caesarean Section 18 years and under	2654
NZ03C	Caesarean Section with complications	3626

^⊥ ^Adapted from HRG4 [41]. ^±^CC Complications. **Cost is expressed in pounds (£) Sterling. ^± ^PbR Payment by Result.
